# Estrogen Receptor β2 Induces Hypoxia Signature of Gene Expression by Stabilizing HIF-1α in Prostate Cancer

**DOI:** 10.1371/journal.pone.0128239

**Published:** 2015-05-26

**Authors:** Prasenjit Dey, Laura A. Velazquez-Villegas, Michelle Faria, Anthony Turner, Philp Jonsson, Paul Webb, Cecilia Williams, Jan-Åke Gustafsson, Anders M. Ström

**Affiliations:** 1 University of Houston, Department of Biology and Biochemistry, Center for Nuclear Receptors and Cell Signaling, 3605 Cullen Boulevard, Science & Engineering Research Center, Bldg 545, Houston, Texas 77204–5056, United States of America; 2 Genomic Medicine Program, Houston Methodist Research Institute, Weill Cornell Medical College, Houston, TX 77030, United States of America; 3 Department of BioSciences and Nutrition, Karolinska Institutet, Novum, S-141 57 Huddinge, Sweden; 4 Science for Life Laboratory, Department of Proteomics and Nanotechnology, KTH—Royal Institute of Technology, 171 21 Stockholm, Sweden; University of Dundee, UNITED KINGDOM

## Abstract

The estrogen receptor (ER) β variant ERβ2 is expressed in aggressive castration-resistant prostate cancer and has been shown to correlate with decreased overall survival. Genome-wide expression analysis after ERβ2 expression in prostate cancer cells revealed that hypoxia was an overrepresented theme. Here we show that ERβ2 interacts with and stabilizes HIF-1α protein in normoxia, thereby inducing a hypoxic gene expression signature. HIF-1α is known to stimulate metastasis by increasing expression of Twist1 and increasing vascularization by directly activating VEGF expression. We found that ERβ2 interacts with HIF-1α and piggybacks to the HIF-1α response element present on the proximal Twist1 and VEGF promoters. These findings suggest that at least part of the oncogenic effects of ERβ2 is mediated by HIF-1α and that targeting of this ERβ2 – HIF-1α interaction may be a strategy to treat prostate cancer.

## Introduction

Prostate cancer is a slowly progressing disease, initially treatable with androgen- deprivation therapy (ADT) [1], but usually recurring in a more aggressive form that is androgen independent [2, 3]. Most aggressive prostate cancers express high levels of androgen receptor (AR) and, in addition, utilize a variety of mechanisms to activate AR in the absence of its ligand. For instance, the cancer can acquire the ability to synthesize AR ligands, phosphorylate AR or, through alternative splicing, create a constitutively active AR [4]. In contrast, expression of the main isoform of estrogen receptor β (ERβ/ESR2), ERβ1, is reduced during prostate cancer progression [5–8]. ERβ1 has been shown to down-regulate the expression of AR, so upon depletion of ERβ1, the expression of AR is substantially increased [9]. In addition, ERβ1 has recently been shown to induce apoptosis in prostate cancer cell lines by activating the FOXO3a/PUMA pathway [10]. ERβ1 has also been shown to inhibit epithelial-to-mesenchymal transition (EMT) by upregulating prolyl hydroxylase domain 2 (PHD2/EGLN1) and subsequently decreasing hypoxia inducible factor 1α (HIF-1α/HIF1A) levels [11, 12]. On the contrary, ERβ splice variant ERβ2 [13] is expressed in late stage metastatic prostate cancer and nuclear ERβ2 expression correlates with decreased overall survival [14]. ERβ2 contains a truncated ligand-binding domain (LBD) and a unique C-terminal amino acid sequence encoded by a unique alternate ERβ2-specific exon called cx [13]. This ERβ isoform lacks the capacity to bind ligand, homodimerize and activate canonical ERβ1 gene expression pathways, but can heterodimerize with ERα thereby inhibiting ERα activity [13]. Nuclear ERβ2 increases invasiveness of PC3 cells [14] and increases cellular proliferation and expression of Twist1 (TWIST1) and c-Myc (MYC) in both PC3 and 22Rv1 cells, indicating possible oncogenic roles of ERβ2 in prostate cancer [15].

A proliferating tumor is often exposed to hypoxic condition, because of its higher metabolic needs and lack of neo-vascularization to keep up with its demands. Hypoxia promotes neuro-endocrine differentiation of the prostate tumor, which increases its aggressiveness [16]. The transcription factor HIF-1α is a central factor in the response of the cells to hypoxia. In cells with normal oxygen levels, HIF-1α is hydroxylated by prolyl hydroxylase, an enzyme that uses oxygen as cofactor and is active only under normoxic condition [17]. Prolyl hydroxylation causes HIF-1α to interact with the Von Hippel Lindau factor (VHL), leading to ubiquitination and degradation of HIF-1α by the proteasome complex [18–21]. Recently, several studies have found that HIF-1α protein can be stabilized without a decrease in oxygen tension by factors interfering with oxygen-dependent HIF-1α degradation [22–24]. After stabilization, HIF-1α translocates to the nucleus and activates transcription of genes involved in angiogenesis. In cancers, HIF-1α changes expression of genes leading to increased tumor metabolism and metastasis, creating a very aggressive tumor. For instance, HIF-1α-dependent regulation of Twist1 expression is a key step in metastasis [25]. Since ERβ2 has a suggested oncogenic role in prostate cancer, and its splice variant ERβ1 is a tumor suppressor known to inhibit HIF-1α, we here investigated whether ERβ2 can increase HIF-1α stabilization, and whether this mechanism underlies the correlation of both factors with aggressive, metastasizing, prostate cancer.

## Materials and Methods

### Reagents and cell culture

The 22Rv1 and PC3 cell lines were obtained from the American Type Culture Collection (ATCC). 22Rv1 cells were maintained in RPMI-1640 (Invitrogen Inc., Carlsbad, CA) medium supplemented with 10% fetal bovine serum (FBS) (Sigma, St. Louis, MO), 25 mM HEPES buffer and 2 mM L-glutamine (Invitrogen Carlsbad, CA), while PC3 cells were maintained in RPMI-1640 (Invitrogen Inc., Carlsbad, CA) medium supplemented with 10% fetal bovine serum (FBS) (Sigma, St. Louis, MO). All experiments used cells below passage 30.

HIG2 luciferase plasmid have been described earlier [26], PXP2-Twist1-LUC and PXP2-mTwist1-LUC have been described [25].

### Construction of an inducible system for ERβ2 and biotin ligase expressing PC3 cells (PC3 BirA)

A transposon-based system mediating doxycycline-regulated expression of ERβ2 was used to stably transfect 22Rv1 and PC3 prostate cancer cells. These cell lines are described elsewhere [15]. For construction of PC3 cells expressing biotin ligase, PC3 cells were transfected with pBirA plasmid expressing *Escherichia coli* biotin holoenzyme synthetase (BirA) from the actin promoter and selected with G418 at 500 μg/ml. After two weeks clones were isolated and assayed for biotinylation activity.

### Protein extract preparation

To prepare whole-cell extracts, cells were washed twice with PBS, lysed in 10 times packed cell volume of lysis buffer [0.1% Nonidet P-40, 250 mM KCl, 5 mM Hepes, pH 7.9, 10% (vol/vol) glycerol, 4 mM NaF, 4 mM sodium orthovanadate, 0.2 mM EDTA, 0.2 mM EGTA, 1 mM dithiothreitol, 1 mM phenylmethylsulfonyl fluoride, protease inhibitor cocktail, PhosStop (Roche, Indianapolis, IN)] for 15 minutes on ice and then centrifuged at 14,000 x *g* for 10 minutes.

### Western blotting

Twenty micrograms of protein were loaded on an SDS-PAGE 10% Bis-Tris gel with Tris running buffer and transferred to a nitrocellulose membrane after electrophoretic separation. Membranes were blocked with 5% non-fat powdered milk in 0.1% TBST buffer and probed with anti-ERβ2 (produced in the lab), Twist1 (sc-15393), and HIF-1α (sc-10790) (Santa Cruz Biotechnology, Inc., Santa Cruz, CA). Primary antibodies were used at 1:200–1000 dilutions, and secondary antibody was used at 1:10,000.

### RNA extraction and real-time PCR

RNA extraction was performed with Qiagen mRNA extraction kit according to standard protocol. cDNA was synthesized from 1μg of total RNA with First Strand System according to standard protocol (Invitrogen Inc. NY). Real-time PCR was performed with SYBR Green I dye master mix (Applied Biosystems Foster City, CA). Primers (Integrated DNA Technologies, Inc. Coralville, IA) were: 18s rRNA forward (F), 5′-CCT GCG GCT TAA TTT GAC TCA-3′ and reverse (R), 5′-AGC TAT CAA TCT GTC AAT CCT GTC C-3; glyceraldehyde-3-phosphate dehydrogenase F, 5′-TGA CAA CTT TGG TAT YCG TGG AAG G-3′ and R, 5′-AGG CAG GGA TGA TGT TCT GGA GAG-3′ (reference genes); ERβ2 F, 5-ACT TGC TGA ACG CCG TGA CC-3 and R, 5′-CCA TCG TTG CTT CAG GCA A-3′; Twist1 F, 5′-GGA GTC CGC AGT CTT ACG AG-3′ and R, 5′-TCT GGA GGA CCT GGT AGA GG-3′. qPCR reactions were performed with a 7500 Fast Real-Time PCR System (Applied Biosystems) using optimized conditions for SYBR Green I dye system: 50 C for 2 minutes, 95 C for 10 minutes, followed by 40–50 cycles at 95 C for 15 seconds and 60 C for 50 seconds. Optimum primer concentration was determined in preliminary experiments, and amplification specificity confirmed by dissociation curve analysis.

### In vitro translation and bacterial expression

HIF-1α and ERβ2 were translated using the TNT Quick Coupled Transcription/Translation Systems (Promega Madison, WI). Briefly, 0.2 μg of HIF-1α or ERβ2 expression vectors (T7 promoter) were added to an aliquot of the TNT Quick master mix and incubated in a volume of 50 μl for 60 minutes at 30°C. The in vitro synthesis of protein was verified by SDS-PAGE. For bacterial expression BL21-DE3 bacterial cells were used to express His-LBD-ERα, His-LBD-ERβ1, His-LBD-ERβ2 and His-LBD-ERβ2ΔCX expression plasmids.

### Pull down from PC3 or HEK293 cells

PC3 cells were transfected with 3 μg BirA (biotin ligase) expression plasmid, 3 μg plasmid containing biotinylation consensus tagged receptors B7TEV-ERα, B7TEV-ERβ1, B7TEV-ERβ2 and B7TEV-ERβ5 together with 3 μg pcDNA3 HA-HIF-1α “Addgene plasmid 18949” or pcDNA3 HA-HIF-1α P405/A-P564/A “Addgene plasmid 18955” or pcDNA3 HA-HIF-1α Δ401–603 (pcDNA3 HA-HIF-1αΔODD) described by Kondo *et al* and Yan *et al*. [27, 28] in a 100 mm tissue culture plate. For HEK293 cells the biotin ligase was transiently transfected. After 48 h, cells were scraped, pelleted and lysed in 300 μl NETN (20 mM Tris (pH = 8.0), 100 mM NaCl, 1 mM EDTA, 0.5% Nonidet P-40), briefly sonicated and centrifuged to remove debris. 10μl streptavidin magnetic beads (Pierce Rockford, IL) were washed in NETN and incubated with 300 μl cellular extract for 2 hours in the cold room. Beads were washed (3 x 10 minutes rotation) with 300 μl NETN and boiled with 20 μl of 2× sample buffer [125 mM Tris HCl (pH 6.8), with 4% SDS, 20% (vol/vol) glycerol, and 0.004% bromophenol blue] and subjected to SDS-PAGE and transferred to nitrocellulose membrane for Western blot. The blot was probed with HIF-1α antibody (Santa Cruz Dallas, TX) 1:1000 dilution and secondary antibody 1:10,000 dilution.

### In-vitro pull downs

2μl *in vitro* translated HIF-1α protein was incubated with 25 μl of bacterial lysate containing His-ERβ2 (LBD) overnight, and complexes were adsorbed onto nickel agarose beads for 2 h. Beads were washed three times with ice-cold NETN (20 mM Tris (pH = 8.0), 100 mM NaCl, 1 mM EDTA, 0.5% Nonidet P-40). Complexes were boiled with 20 μl of 2× sample buffer, subjected to SDS-PAGE and transferred to nitrocellulose membrane for Western analyses. The blot was probed with anti-HIF-1α antibody (Santa Cruz). Primary antibodies were at 1:1000 dilution, and secondary antibody at 1:10,000.

### Mammalian 2 hybrid assay

ERβ2 was cloned into the Gal4DBD expressing plasmid PM2 in frame with the Gal4DBD domain using BamHI and HindIII restriction sites making PM2-ERβ2. The pVP16 plasmid (Clontech) was used to clone the N-terminal HIF-1α (amino acids 1–401), oxygen destabilization domain (amino acids 401–603) and the C-terminal domain (amino acids 603–826) in frame with the VP16 activation domain using BamHI and PstI restriction sites. For interaction assays in PC3 cells the Gal4 responsive reporter FR-LUC 300 ng, PM2-ERβ2 500 ng and VP16 fused HIF-1α domains 200 ng was transfected using PEI method of transfection as described by Longo et al. [29]

### Microarray and bioinformatic analyses

The two-color comparative microarray analysis covers the fully known protein-coding transcriptome of 39,600 transcripts and variants by using Human OpArray arrays purchased from Microarrays Inc. (Huntsville, AL). The array was complemented with 133 oligonucleotides specifically synthesized for detailed and robust analysis of nuclear receptors, splice variants, and coregulators. The microarray analysis was performed essentially as in Richter *et al*. [30]. For each cDNA synthesis, 20 μg of total RNA were used, and each comparison was replicated and dye swapped. Bioinformatic analyses were performed using GenePix, R, and Pathway Studio software (Elsevier, Philadelphia, PA). Differentially expressed genes were defined using a p-value cut-off of p = 0.005 in combination with an M-value (log2 of fold-change) cut-off of +/- 0.3. Enrichment analysis of gene ontology (GO) of the biological processes was performed with Fisher's exact test using the software's gene sets. Pathway pictures were designed in Pathway Studio. Microarray data is available in NCBI's Gene Expression Omnibus under accession number GSE35095.

### Chromatin Immunoprecipitation (ChIP)

Sub-confluent PC3 and 22Rv1 cells (90%) were grown in a 150 mm dish and washed twice with cold PBS and then fixed in PFA (1%) for 10 minutes. Cells were washed twice with cold PBS + protease inhibitor, scraped with 150 μl of resuspension buffer, and centrifuged (4000rpm, 10 minutes). Pellets were resuspended in 750 μl ChIP lysis buffer (HEPES 50 mM, NaCl 140 mM, triton 1%, protease inhibitor) and cells kept on ice for 30 minutes. Cell lysates were sonicated (60 pulses, 30 seconds each) with 30 seconds break at 4°C. Following sonication, 25 μl samples were collected and stored at -20°C (input). The rest of the 125 μl cell lysate fraction was used for the following steps. Samples were pre-absorbed in 20 μl of G protein coupled FLAG tagged magnetic beads at 4°C for 2 hours and supernatant collected. Samples were then divided into two fractions and antibodies against FLAG (2.5 μg) or IgGs (2.5 μg) were added and incubated overnight. The next day, the beads were washed three times with ChIP lysis buffer, once with ChIP wash high salt buffer (HEPES 50 mM, NaCl 500 mM, triton 1%), and finally once with ChIP wash buffer (Tris 10 mM, LiCl 250 mM, NP40 0.5%, EDTA 0.1 mM). Beads were resuspended in 40 μl elution buffer (Tris 50 mM, SDS 1%, EDTA 10 mM) and incubated at 65°C overnight with a small hole on the cap. The input collected previously was also placed at 65°C. The next day, samples were purified using PCR purification kit (Qiagen) and used for qPCR analysis using the following primers. pHRE-Twist1 promoter (F) 5’-CGGGGGAGGGGGACTGGAAAGC-3’; (R) 5’-AGGCCTCCTGGAAACGGTGCCG-3’, VEGF promoter: (F) 5’-ACAGACGTTCCTTAGTGCTGG-3’; (R) 5’-AGCTGAGAACGGGAAGCTGTG-3’.

### Statistics

The values are expressed as the mean with 95% confidence intervals. An unpaired two-tailed *t*-test was used to compare the differences between two groups. The significance is presented as *p<0.05, **p<0.005, and ***p<0.001, and non-significant differences are presented as NS.

## Results

### Microarray and bioinformatic analysis of prostate cancer cells expressing ERβ2 show increased expression of genes involved in hypoxic response

We have previously shown that ERβ2 increased expression of EMT-associated genes Twist1 and Slug (SNAI2) in prostate cancer cells [15]. Here, we performed a transcriptomic study to explore the effects of ERβ2 in an unbiased manner. We compared PC3 cells stably expressing ERβ2 with control cells, and found 585 genes to be significantly changed using our defined cut-off. Interestingly, TGFB2, which is known to be stimulated by HIF-1α, was among the most highly upregulated genes (almost 9-fold, according to microarray). Analyzing enrichment of gene ontology categories among the regulated genes, ‘*response to hypoxia*’ was the second most overrepresented function (p = 8.0e-6), second only to ‘gene expression’ (Table 1). The ‘*Response to hypoxia*’ group included 17 significantly regulated genes (TGFB2, EGLN1 (PHD2), EGLN3 (PHD3), SMAD3, HSD11B2, CAT, CCL2, CDKN1B, MMP13, PLOD2, IL1B, PLAU, SCNN1G, CRYAB, IL18, ITPR1, SOD2, ADM). In addition, the enriched sub-network, defined as the genes being expression target of the respective node, ‘Neighbors of HIF-1α’ was the most overrepresented one (p = 1.5e-09) with 41 regulated members (Table 2). Further, many ERβ2-induced genes were found to be involved in angiogenesis (Table 2), which was also an overrepresented theme (p < 0.05). Of these genes, SOD2, SCNN1G, CD24, HIG2, IGF2 and TGF-β2 are all stimulated by HIF-1α [26]. Also ‘*glucose metabolism process’* which HIF-1α is known to regulate, was overrepresented (p < 0.007), including 9 genes (PFKP, AKT1, IGF2, GOT2, PGM5, CRYAB, GBE1, UGP2, FABP5). Thus, the microarray analysis clearly indicated hypoxia and HIF-1α function as major themes of ERβ2 function (See S1 Fig for validated genes).

**Table 1:** Gene functions overrepresented in ERβ2 expressing PC3 cells.

Gene Ontology-Biological Process	Genes Regulated	Percent-of-genes Included in function	P—Value
Gene expression	32	4	1.80E-06
Response to hypoxia	17	6	8.00E-06
Small molecule metabolic process	44	3	0.000089
Cellular protein metabolic process	20	4	0.00011
Negative regulation of cell proliferation	20	4	0.00024
Ribosome biogenesis	8	9	0.00024
Activation of cysteine-type endopeptidase activity involved in apoptotic process	8	8	0.00033
Apoptotic mitochondrial changes	4	21	0.00039
Glycogen biosynthetic process	4	20	0.00048
Positive regulation of lipid biosynthetic process	3	33	0.00052
Protein folding	12	5	0.00059
Glucose metabolic process	9	7	0.00067

**Table 2:** ERβ2 regulation of hypoxic genes in PC3 cells.

Response	Genes	P-value
Response to hypoxia	PLAU, SOD2, SCNN1G,CD24,HIG2, TGF-β2, EGLN3	<0.001
Angiogenesis	SOD2, SCNN1G, CD24, HIG2, IGF2, TGF-β2, ILB, PLAU, FGFR1, HOXB13, SRPX2	<0.05
Glucose metabolism process	PFKP, AKT1, IGF2, PGM5, CRYAB, GBE1, UGP2, FABP5	<0.007
VEGF production	IL1B, C3	<0.001
Blood vessel morphogenesis	PLAU, FGFR1	<0.001

**Fig 1:**
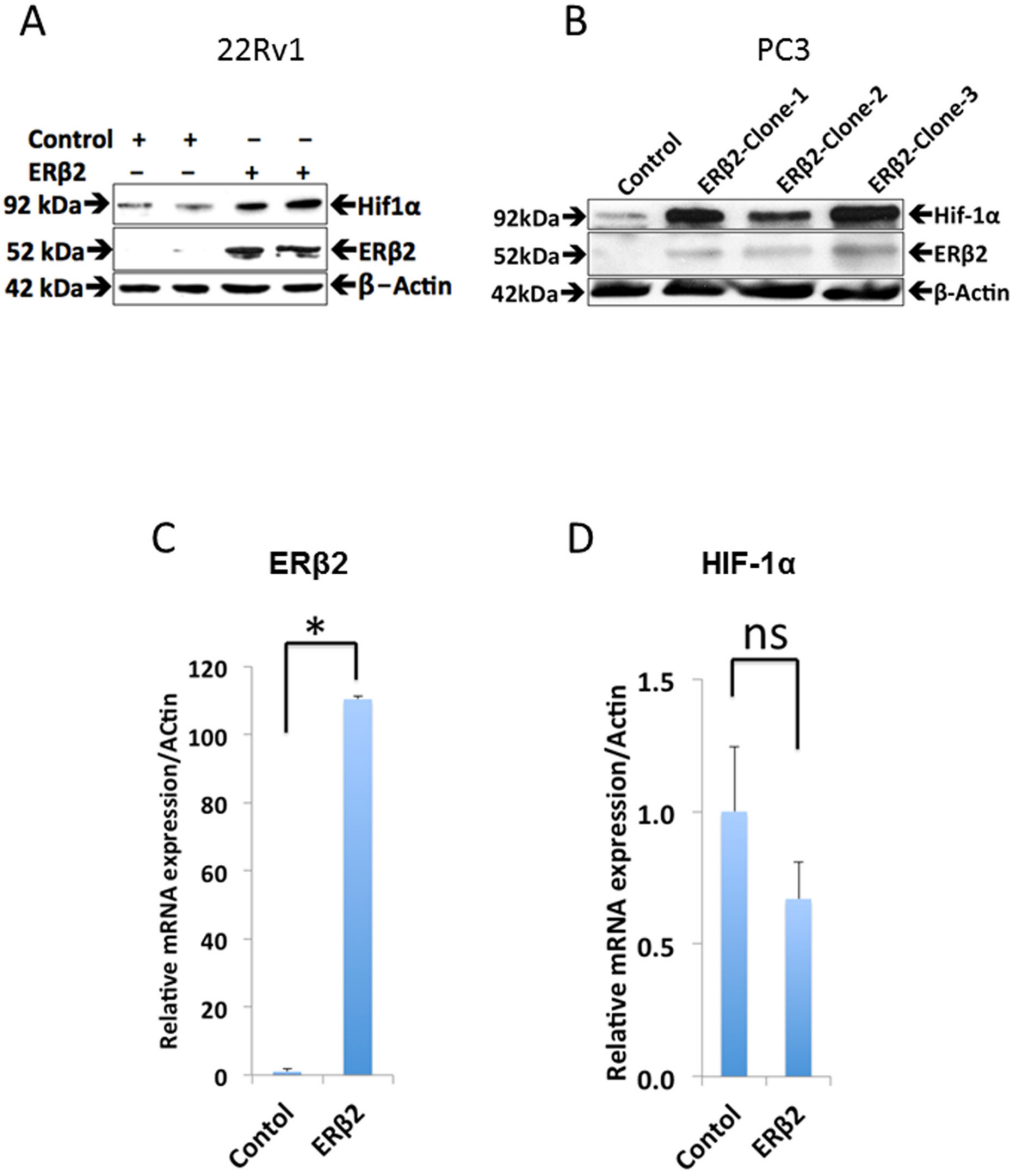
Regulation of HIF-1α protein expression independent of mRNA regulation. A. Western blot of extracts from control and two different 22Rv1 and PC3 clones expressing ERβ2 assayed with HIF-1α antibody. B. Relative mRNA expression of ERβ2 and HIF-1α in the ERβ2clones of the 22Rv1 cell line. The graph shows the data as fold change compared with the control (mean of three separate experiments (±s.e.m.) calculated using Student’s *t*-test, *p≤0.016). C. Relative mRNA expression of HIF-1α in the ERβ2 #2 clone of the 22Rv1 cell line. The graph shows the data as fold change compared with the control (mean of three separate experiments (±s.e.m.) calculated using Student’s *t*-test, **p≤0.006).

**Fig 2:**
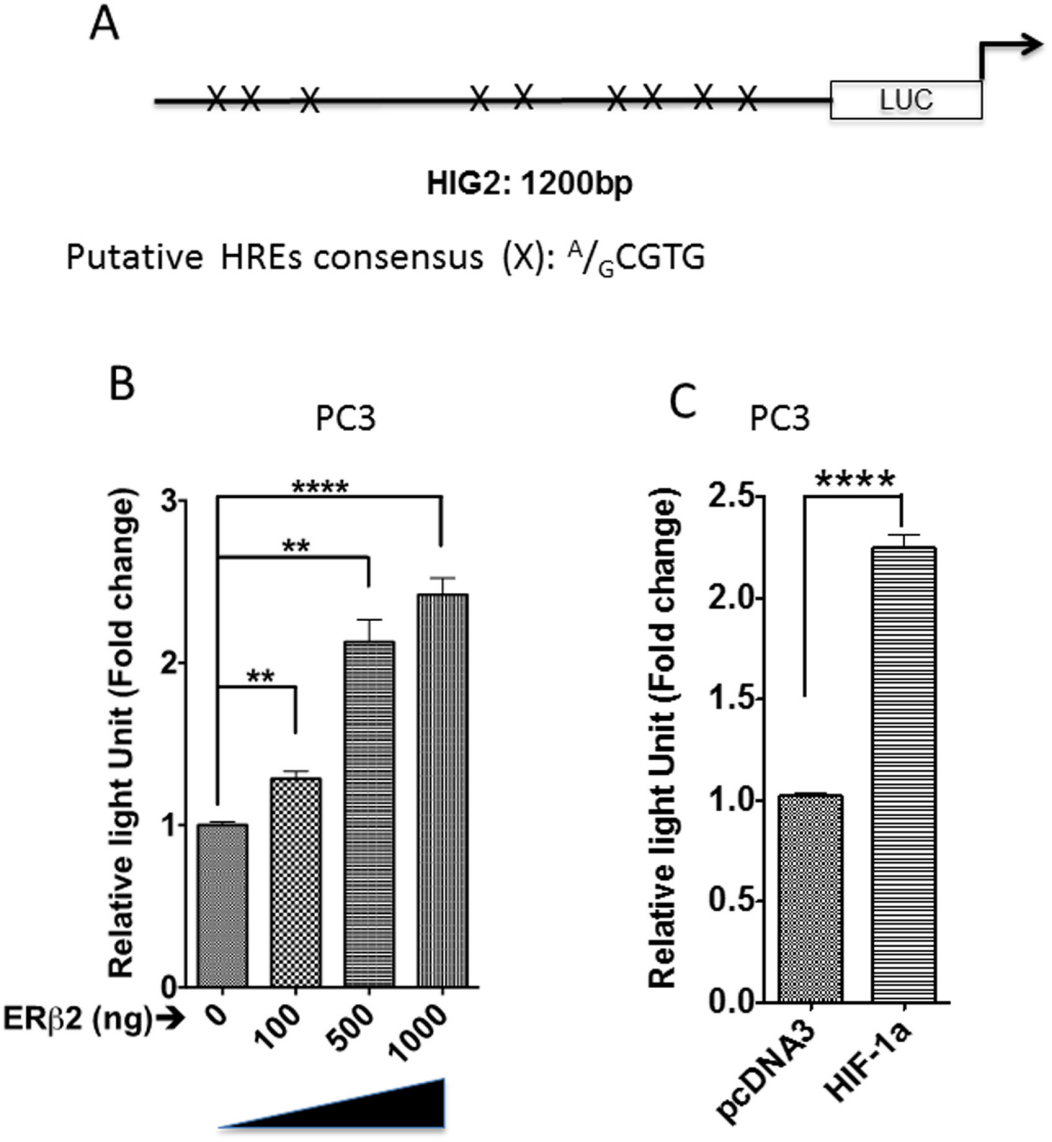
HIG2 promoter is activated by ERβ2 expression. A. Schematic outline of the HIG2 promoter construct where (X) is representing sequence of HIF-1α response elements (HRE). B. Increasing amount of transient transfected ERβ2 expression plasmid into PC3 cells successively activates the transiently transfected HIG2 promoter as shown by luciferase assay of HIG2-A-LUC. The graph shows the data as an increase in luminescence of cells transfected with increased concentration of ERβ2 (mean of three separate experiments (±s.e.m.) calculated using Student’s *t*-test, ***p≤0.0002). Comparison of concentrations—0μg to 1000 μg. C. Addition of expression plasmid for HIF-1α activates HIG2 promoter in PC3 cell lines. The graph shows the data as an increase in luminescence of cells transfected with HIF-1α (mean of three separate experiments (±s.e.m.) calculated using Student’s *t*-test, ****p≤0.0001).

**Fig 3:**
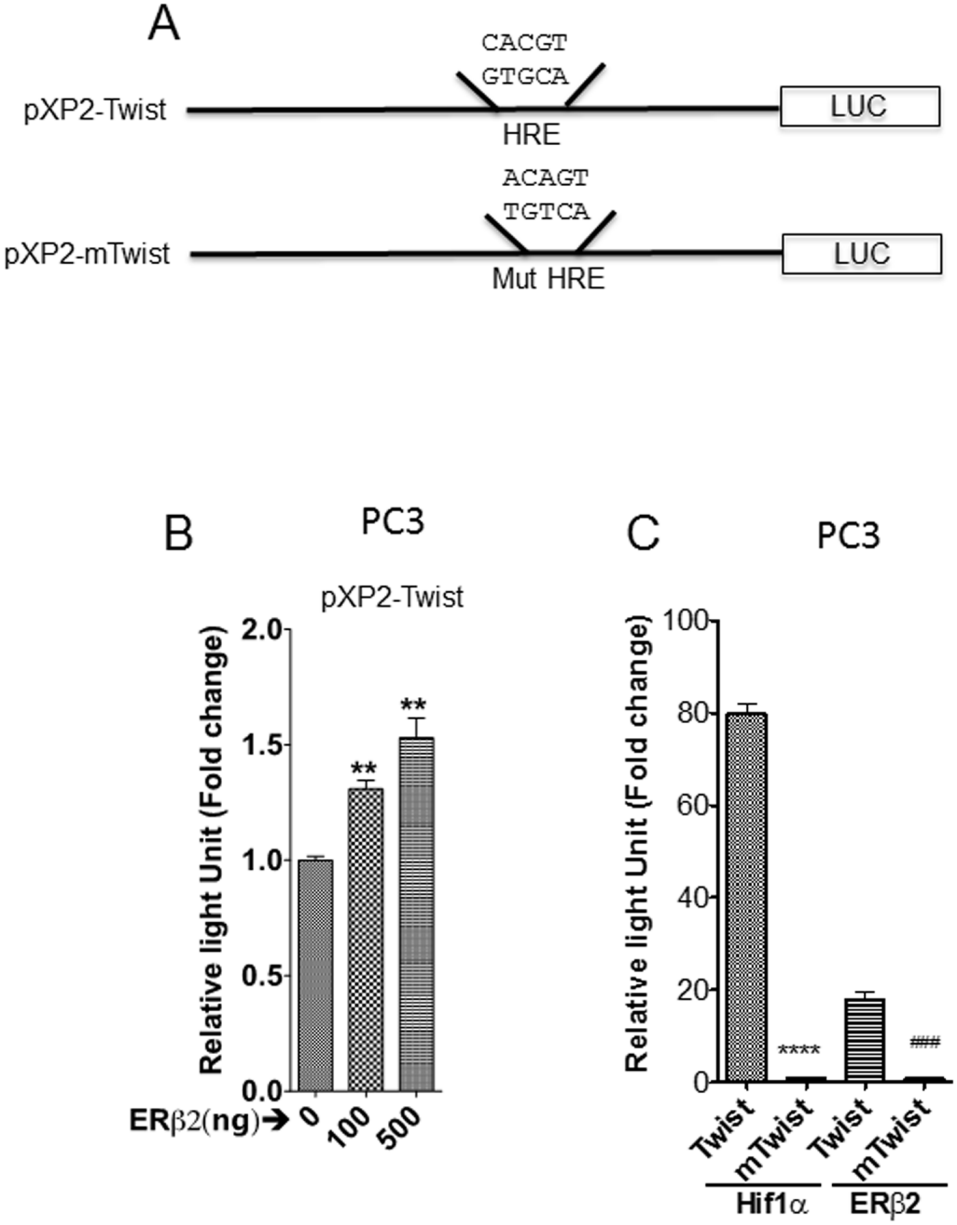
Twist1 promoter with intact HIF-1α response element is activated by ERβ2 expression while the promoter with mutated site is not activated. A. Description of Twist1 promoter constructs used. B. LNCaP cells transfected with Twist1-promoter-Luciferase reporter and increasing concentration of ERβ2 expression plasmid. The graph shows the data as an increase in luminescence of cells transfected with increased concentration of ERβ2 (mean of three separate experiments (±s.e.m.) calculated using Student’s *t*-test, **p≤0.0034). C. Transfection of Twist1-promoter-Luciferase reporter with (Twist) or mutated (mTwist) HIF-1α response element together with expression plasmids for HIF-1α or ERβ2 in LNCaP cells. The graph shows the data as an increase in luminescence of cells transfected with increased concentration of ERβ2 (mean of three separate experiments (±s.e.m.) calculated using Student’s *t*-test, ****p≤0.0001, ###p≤0.002).

To validate these data, we analyzed the expression of HIF-1α in PC3 and another prostate cancer cell line-22Rv1, which are both over-expressing ERβ2. We observed an increase in HIF-1α protein level in ERβ2 expressing versus control cells, but no changes in corresponding mRNA level (Fig 1A–1D). This agrees with our microarray results, which did not detect change in HIF-1α transcription levels, but indicated a change in HIF-1α functions (based on enriched pathways). Further, ERβ2-dependent increase in HIF-1α protein levels was confirmed to be accompanied by ERβ2-dependent increase in HIF-1α activity. Luciferase activity of a reporter driven by the HIF-1α-dependent hypoxia inducible gene 2 (HIG2/HILPDA) promoter (HIG2-A-luc) [26] was increased in the presence of transfected ERβ2 expression vector (Fig 2A–2C). In a previous study, we showed that ERβ2-expressing PC3 cells have increased level of Twist1 [15]. Here, we show that ERβ2 also increased activity of a luciferase reporter driven by the HIF-1α-dependent Twist1 promoter in LNCaP prostate cancer cells and that this effect was dependent upon the HIF-1α response element (Fig 3A–3C). This demonstrates that HIF-1α activity in general is enhanced in prostate cells expressing ERβ2.

### ERβ2 directly interacts with HIF-1α causing stabilization

**Fig 4:**
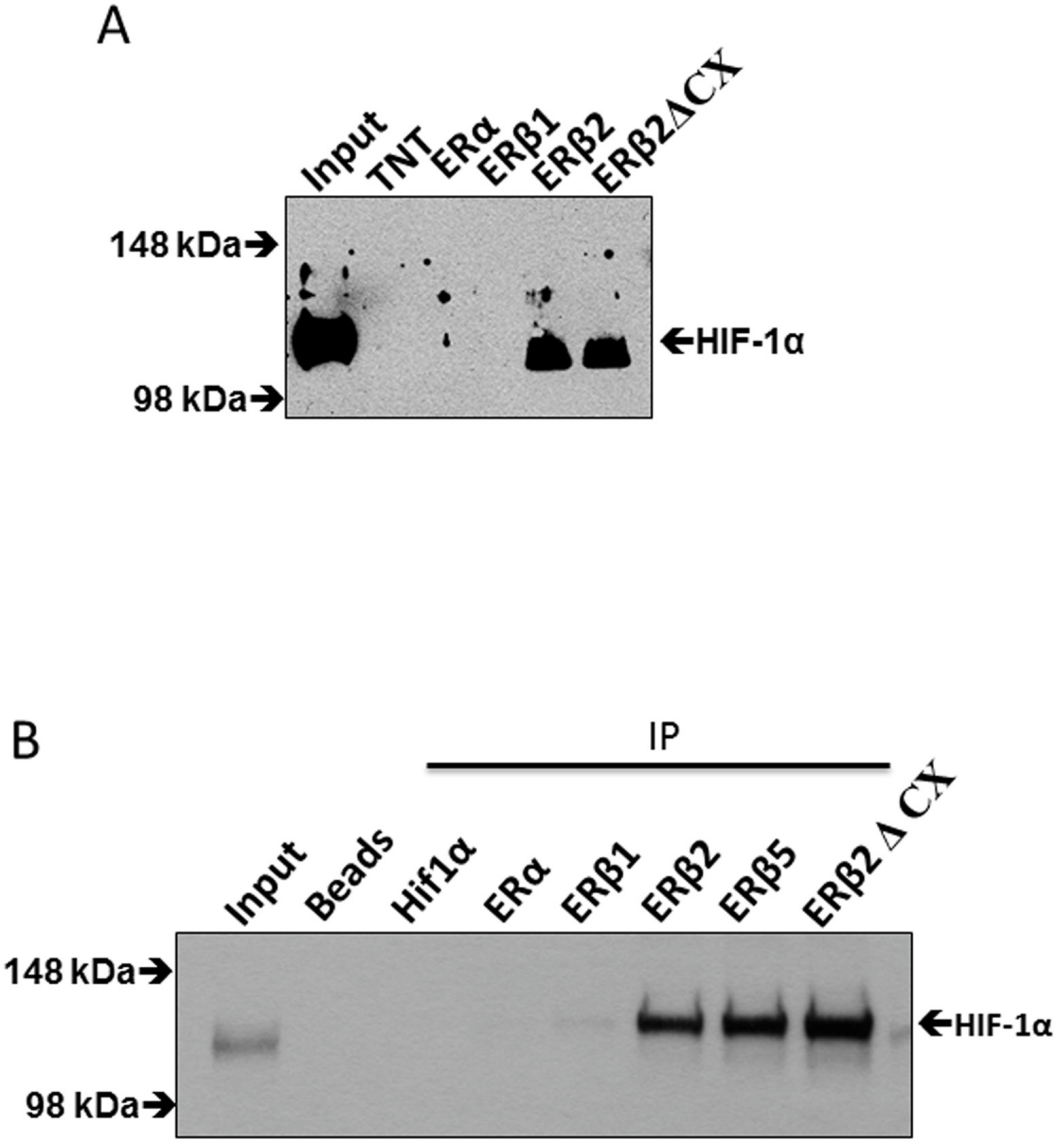
Interaction of HIF-1α with ERβ2 *in vitro*. A. His-tagged LBD-ERβ2 pull down with nickel agarose beads of *in vitro* translated HIF-1α compared to His-tagged LBD-ERα, His-tagged LBD-ERβ1, His-tagged LBD-ERβ2 and His-tagged ERβ2ΔCX TNT is lane with only coupled reticulocyte lysate. B. Expression of the biotin ligase BirA, HA-HIF-1α, and the N-terminal biotin consensus peptide fused receptor constructs B7TEV-ERα, B7TEV-ERβ1, B7TEV-ERβ2, B7TEV-ERβ5, and B7TEV-ERβΔCX in PC3 cells. Cell extracts were then subjected to pulldown with streptavidin magnetic beads and proteins separated by SDS-PAGE followed by detection with HIF-1α antibody.

Since ERβ2 increases expression of HIF-1α protein but not mRNA, we speculated that HIF-1α stabilization occurs through protein-protein interaction between ERβ2 and HIF-1α. To test this hypothesis, we attached bacterially expressed LBDs of ERα, ERβ and ERβ2 to a solid support and determined interactions with *in vitro* translated HIF-1α. HIF-1α strongly bound to ERβ2, but not to equivalent amounts of wild type ERα or ERβ LBDs. This interaction was independent of ERβ2 specific sequences because truncation of amino acids encoded by the ERβ2-specific exon did not eliminate HIF-1α binding (ERβ2ΔCX) (Fig 4A). Pull-down of biotin-tagged ERs expressed in PC3 cells revealed specific interaction of transfected HIF-1α with ERβ2 and ERβ2ΔCX and only modest interaction with ERβ1 (Fig 4B). Since ERβ5 has the same amino acid sequence and is truncated at the same amino acid as ERβ2, i.e. only the small C-terminal peptides differs between the two variants we decided to test this variant and investigate its interaction with HIF-1α. As shown in Fig 4B, ERβ5 interacts strongly with HIF-1α.

### The oxygen destabilizing domain (ODD) of HIF1α is not required for interaction with ERβ2 and ERβ5

**Fig 5:**
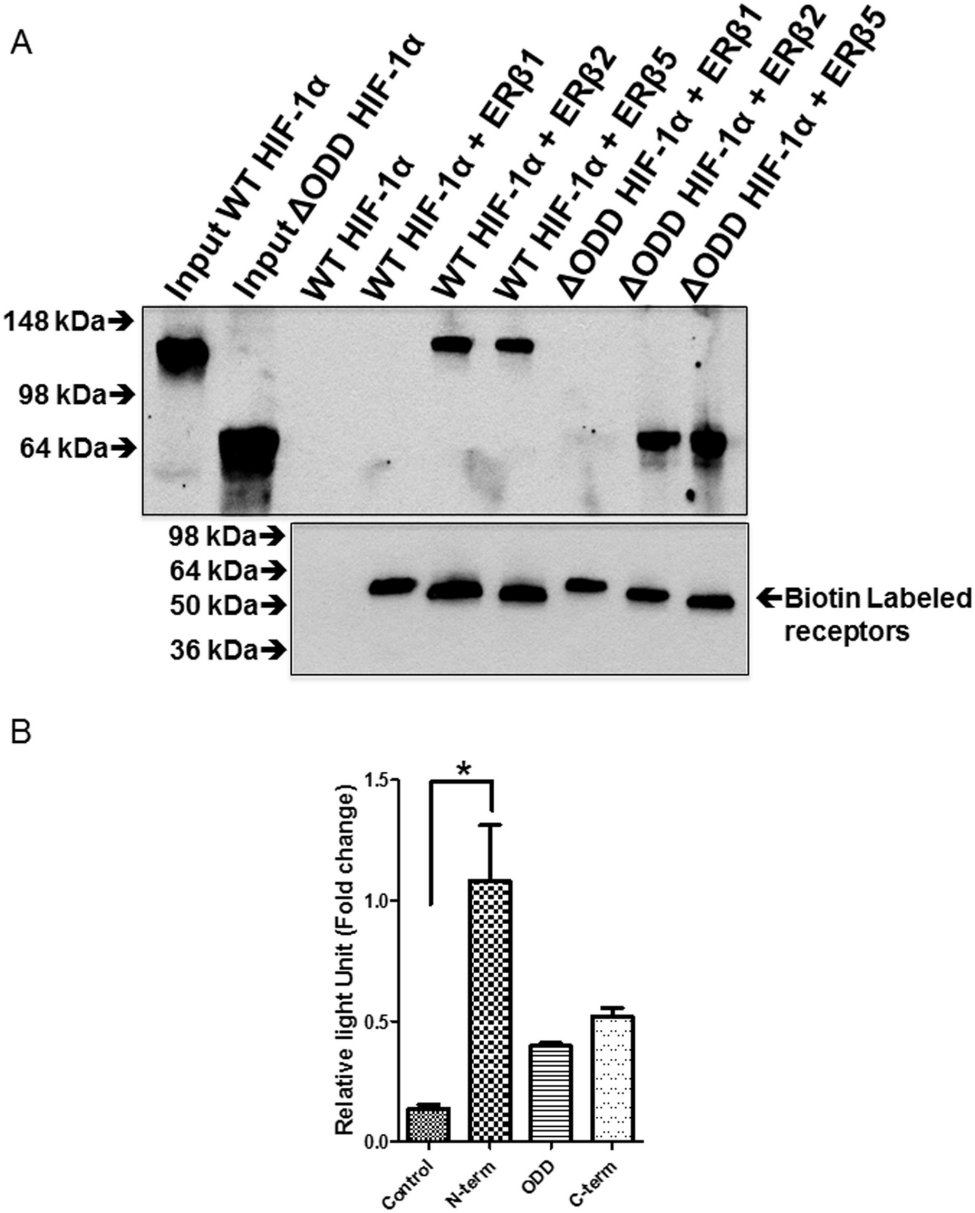
The oxygen destabilization domain is not required for interaction with ERβ2 and ERβ5. A. PC3 cells transfected with plasmids expressing GFP, HIF-1α, HIF-1α ΔODD, BirA (biotin ligase), ERβ2 and ERβ5 with N-terminal biotinylation consensus. Complexes were pulled down with streptavidin magnetic beads. Pulled down HIF-1α and HIF-1α ΔODD was detected using anti-HIF-1α antibody. B. Transfection of 200 ng of FR-luciferase alone, with 500ng PM2-ERβ2 and 300ng of either VP16-N-term HIF-1α (amino acids 1–401), VP16- ODD HIF-1α (amino acids 401–603), or VP16-C-term HIF-1α (amino acids 603–826). Measurement of luciferase activity 24 h after transfection.

To investigate, if the oxygen destabilizing domain of HIF-1α was required for interaction with ERβ2 we constructed an expression plasmid of HIF-1α with amino acids 401–603 deleted as described earlier [31]. We then co-immunoprecipitated this domain deleted HIF-1α with biotinylated ERβ2 and ERβ5. As can be seen in Fig 5A the HIF-1α with deleted ODD (Δ401–603) interacts as strongly as the wild-type indicating that the ODD domain is dispensable for the interaction. To map the interaction further we used mammalian two-hybrid assay where the N-terminus, ODD domain and C-terminus of HIF-1α were fused to VP16 and transfected into PC3 cells together with Gal4DBD fused ERβ2 and a reporter with Gal4 binding sites in front of luciferase. As can be seen in Fig 5B the interaction occurs preferably with the N-terminus of HIF-1α.

**Fig 6:**
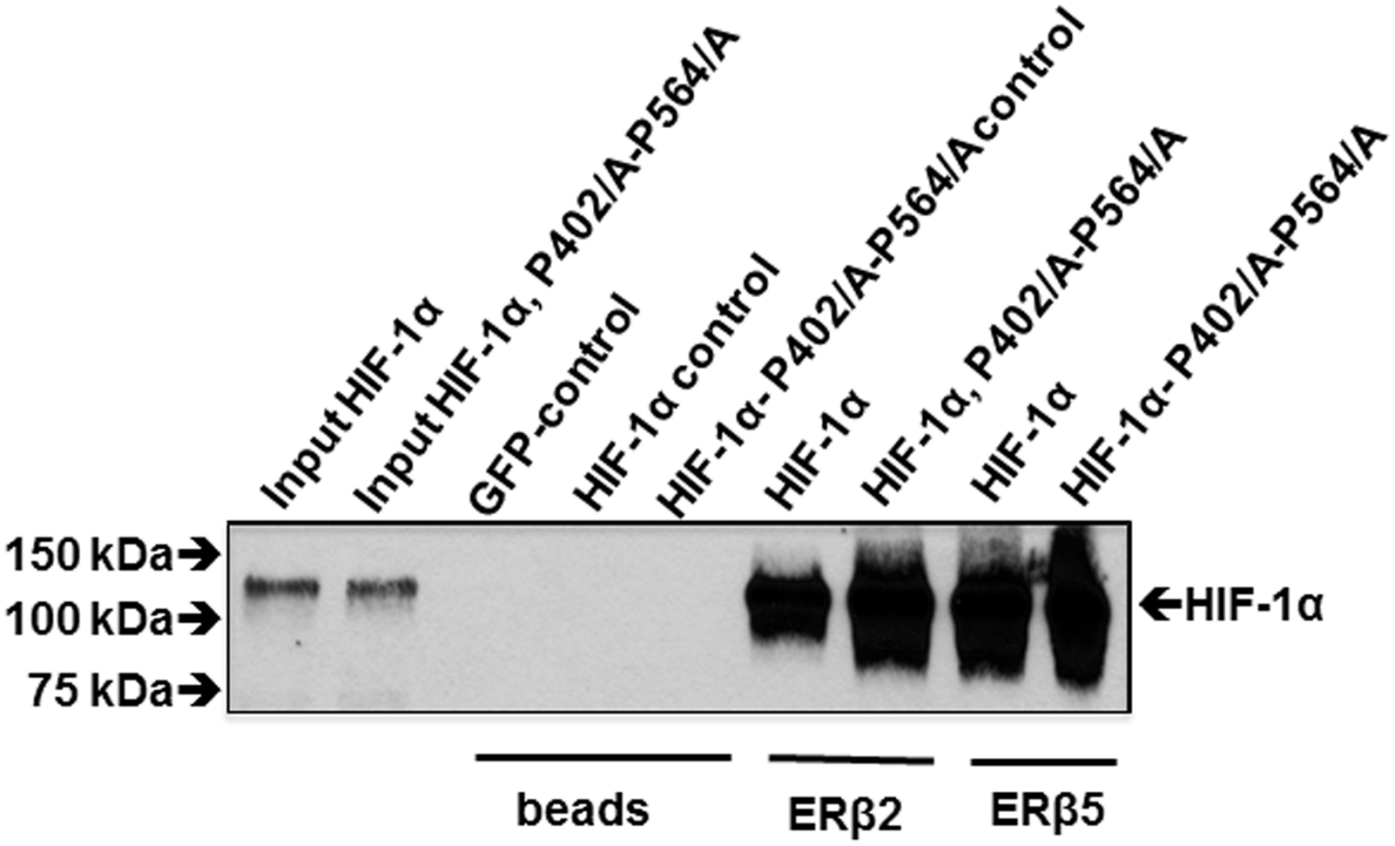
The prolines P405 and P564 are not required for interaction with ERβ2 and ERβ5. PC3 cells transfected with GFP, HIF-1α, HIF-1α P405/A-P564/A, BirA (biotin ligase), ERβ2 and ERβ5 with N-terminal biotinylation consensus. Complexes were pulled down with streptavidin magnetic beads.

It is well known that prolines 405 and 564 of HIF-1α under normoxia are hydroxylated by proline hydroxylases (PHD’s) and then recognized by the VHL factor and subsequently targeted for degradation [32]. We wanted to find out if ERβ2 and ERβ5 interaction with HIF-1α is dependent on hydroxylation of these prolines so that the interaction would even occur during normoxic conditions. In Fig 6, we showed that WT and P405/A–P564/A mutated HIF-1α interact strongly with ERβ2 irrespective of destruction of prolyl hydroxylation sites suggesting that the interaction occurs both during normoxia and hypoxia.

### ERβ2 is recruited to HIF-1α response elements of the Twist1 and VEGF promoters

**Fig 7:**
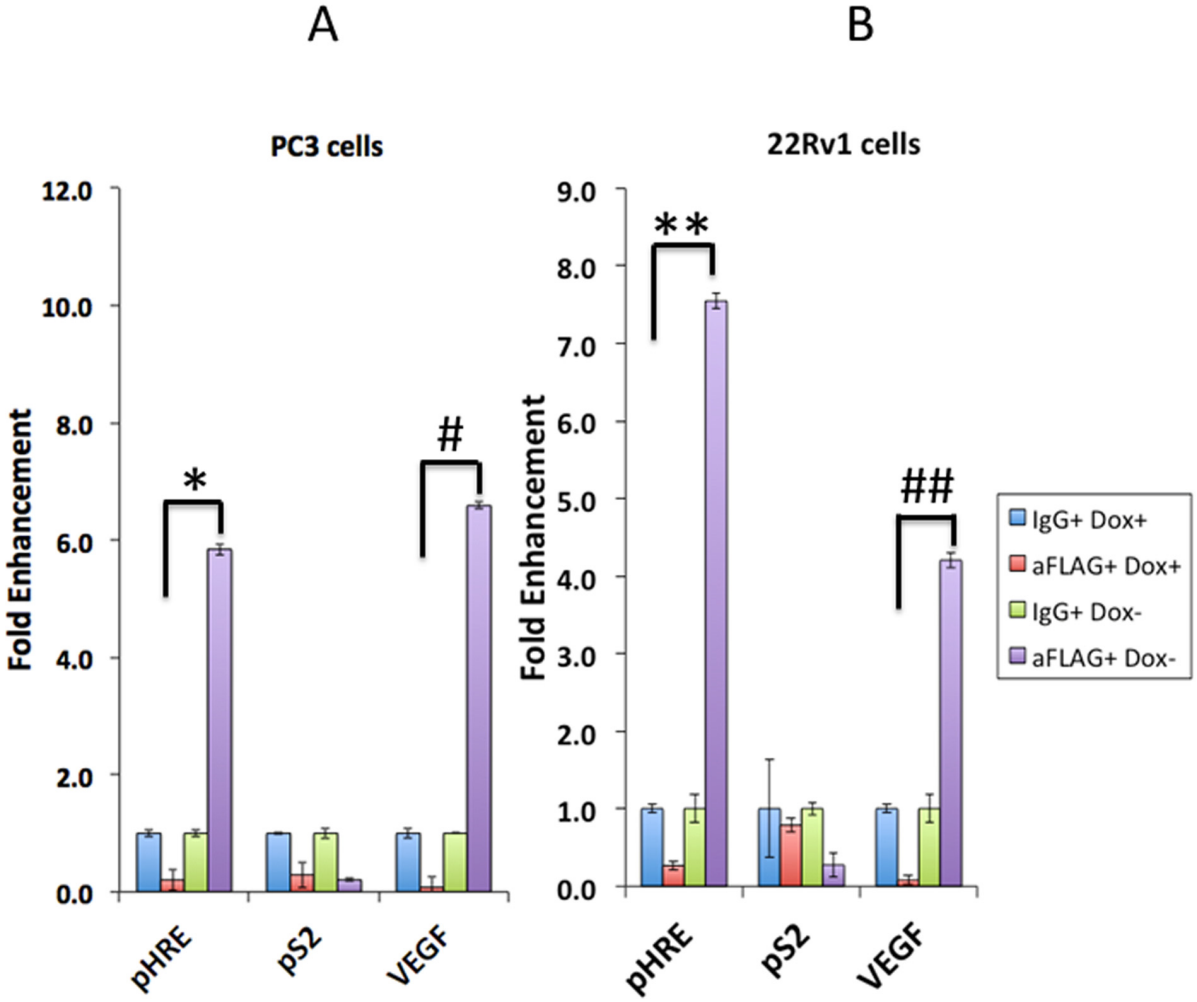
ChIP assay showing recruitment of ERβ2 to the HIF-1α binding site in the Twist1 and VEGF promoters. In PC3 cells (A) and 22Rv1 cells (B) expressing doxycycline-regulated ERβ2 expression, this ERβ isoform binds to HRE (HIF-1α response element in Twist1 promoter) and VEGF promoter (HIF-1α response element in VEGF promoter) but not to pS2 (ERE) promoter. ChIP-qPCR results are shown with a non-specific IgG and a specific anti-M2-FLAG antibody immunoprecipitation. ERβ2 binding is enriched only in HIF-1α response element containing Twist1 and VEGF promoters but not in the pS2 promoter lacking HIF-1α response element. The graph shows the data as an increase in binding of FLAG to the Twist1 (pHRE) and VEGF promoter (mean of three separate experiments (±s.e.m.) calculated using Student’s *t*-test, *p≤0.028, ##p≤0.041 (PC3 cells) and **p≤0.0049, ##p≤0.0481 (22Rv1 cells).

Finally, we explored whether ERβ2 can bind to the HIF-1α response elements using ChIP-qPCR. We detected increased levels of ERβ2 recruited at the HIF-1α response elements on the HIF-1α dependent Twist1 and VEGF promoters in both PC3 and 22Rv1 cells. However, ERβ2 does not bind to the promoters of the classical estrogen response element in the pS2 gene (Fig 7A and 7B).

In conclusion, our findings indicate that ERβ2 binds to and stabilizes HIF-1α and furthermore, is co-recruited to key HIF-1α elements in HIF-1α regulated genes thereby enhancing the activity of HIF-1α during normoxic conditions in prostate cancer cells.

## Discussion

Response of normal tissues to hypoxia is crucial for proper vascularization and for subsequent blood supply to oxygenate and provide nutrients to the tissue. A growing tumor becomes hypoxic when reaching more that 1mm in size [33]. The immediate response to hypoxia is a decreased prolyl-hydroxylation of HIF-1α leading to increased stabilization of this protein. This is because VHL, which induces ubiquitinylation and subsequent degradation of HIF-1α under normoxic conditions, cannot bind to HIF-1α in the absence of prolyl-hydroxylation. Stabilization of HIF-1α allows regulation of genes involved in angiogenesis such as VEGF, which is secreted from the tumor, attracting endothelial cells by binding to their surface VEGF receptors thus allowing increased tumor vascularization and growth. HIF-1α also increases expression of genes involved in glycolysis, to adapt the tumor to survive under conditions of low oxygen [34], and genes involved in invasion and metastasis such as Twist1 [25].

Expression of ERβ2 correlates with worse prognosis in prostate cancer [14] and ERα-negative breast cancer [35], but possible oncogenic actions of ERβ2 are not understood. We show here that there is a strong correlation between ERβ2 expression and HIF-1α protein level but not mRNA level in the prostate cancer cell lines PC3 and 22Rv1. ERβ2 enhances the activity of HIF-1α dependent promoters and is recruited to the Twist1 and VEGF promoters, likely by tethering to HIF-1α. Together, the findings outlined above suggest that HIF-1α mediates the oncogenic effect of ERβ2 through a non-classical signaling pathway in which ERβ2 binds to, and stabilizes HIF-1α under normoxic conditions. This is in line with other studies showing that HIF-1α interacting proteins stabilize HIF-1α by blocking its degradation [22–24].

Surprisingly, although we found that ERβ1 does not interact efficiently with HIF-1α, correlating with its inability to induce HIF-1α responsive genes, the unique last exon of ERβ2 is not required for HIF-1α binding. Apparently, truncation of the ERβ1 C-terminus exposes a new protein surface mediating interaction with HIF-1α.

**Fig 8:**
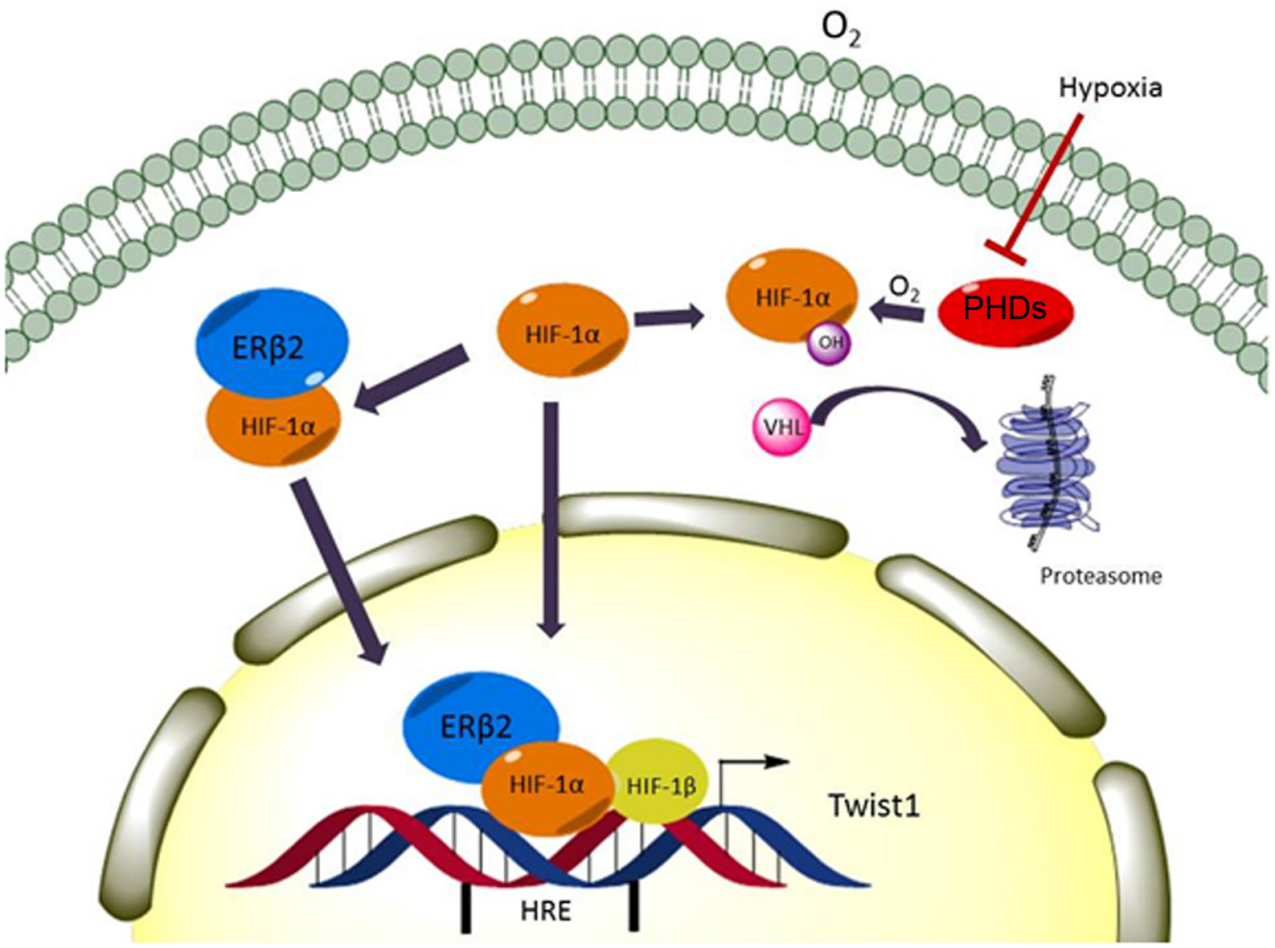
Schematic model of the interaction between ERβ2 and HIF-1α. Proposed model of how ERβ2 stabilizes HIF-1α and is recruited to VEGF and Twist1 promoters.

It remains to be shown if ERβ2 expression correlates with levels of Twist 1 or other HIF-1α target genes in clinical samples. A recent study by Ragnum et al. [36] shows that androgen-deprivation therapy (ADT) causes a reduced expression of many genes linked to hypoxia. We propose that expression of ERβ2 or ERβ5 could counteract the effect of ADT on hypoxia-linked genes in a prostate tumor and thus cause castration resistance resulting in a poorer outcome. Indeed, our findings of non-hypoxic stabilization of HIF-1α suggest that it might be possible to develop a small molecule interfering with the ERβ2 and ERβ5-induced HIF-1α stabilization for use in therapy of certain aggressive forms of prostate cancer. An overview of the described findings is shown in Fig 8.

In conclusion, expression of the ERβ variants ERβ2 and ERβ5 has previously been shown to correlate with aggressive prostate cancer. Here we show that ERβ2 and ERβ5 stabilize the HIF-1α protein and induce hypoxic gene expression under normoxic conditions, proposing a mechanism for the oncogenic effect of ERβ2 and ERβ5.

## Supporting Information

S1 Fig A. Western blot for HIF-1α on extracts from control 22Rv1 cells and two clones expressing ERβ2. (B) Validation of genes from the microarray.
(TIF)
