# Correction: Estrogen Receptor β2 Induces Hypoxia Signature of Gene Expression by Stabilizing HIF-1α in Prostate Cancer

**DOI:** 10.1371/journal.pone.0132085

**Published:** 2015-06-26

**Authors:** Prasenjit Dey, Laura A. Velazquez-Villegas, Michelle Faria, Anthony Turner, Philip Jonsson, Paul Webb, Cecilia Williams, Jan-Åke Gustafsson, Anders M. Ström

The fifth author’s name is spelled incorrectly. The correct name is: Philip Jonsson.

The affiliation for the fifth author is incorrect. Philip Jonsson is not affiliated with #1 but with #5: Department of Radiation Oncology, Center for Molecular Oncology, Memorial Sloan Kettering Cancer Center, NY, 10022, USA

## References

[1] Dey P, Velazquez-Villegas LA, Faria M, Turner A, Jonsson P, Webb P, et al (2015) Estrogen Receptor β2 Induces Hypoxia Signature of Gene Expression by Stabilizing HIF-1α in Prostate Cancer. PLoS ONE 10(5): e0128239 doi: 10.1371/journal.pone.0128239 26010887 10.1371/journal.pone.0128239 PMC4444278

