# Supplementary figures and images for: Estrogen Receptor β2 Induces Hypoxia Signature of Gene Expression by Stabilizing HIF-1α in Prostate Cancer
Source: PLoS One. 2015 May 26;10(5):e0128239. doi: 10.1371/journal.pone.0128239 (PMC4444278; doi:10.1371/journal.pone.0128239)

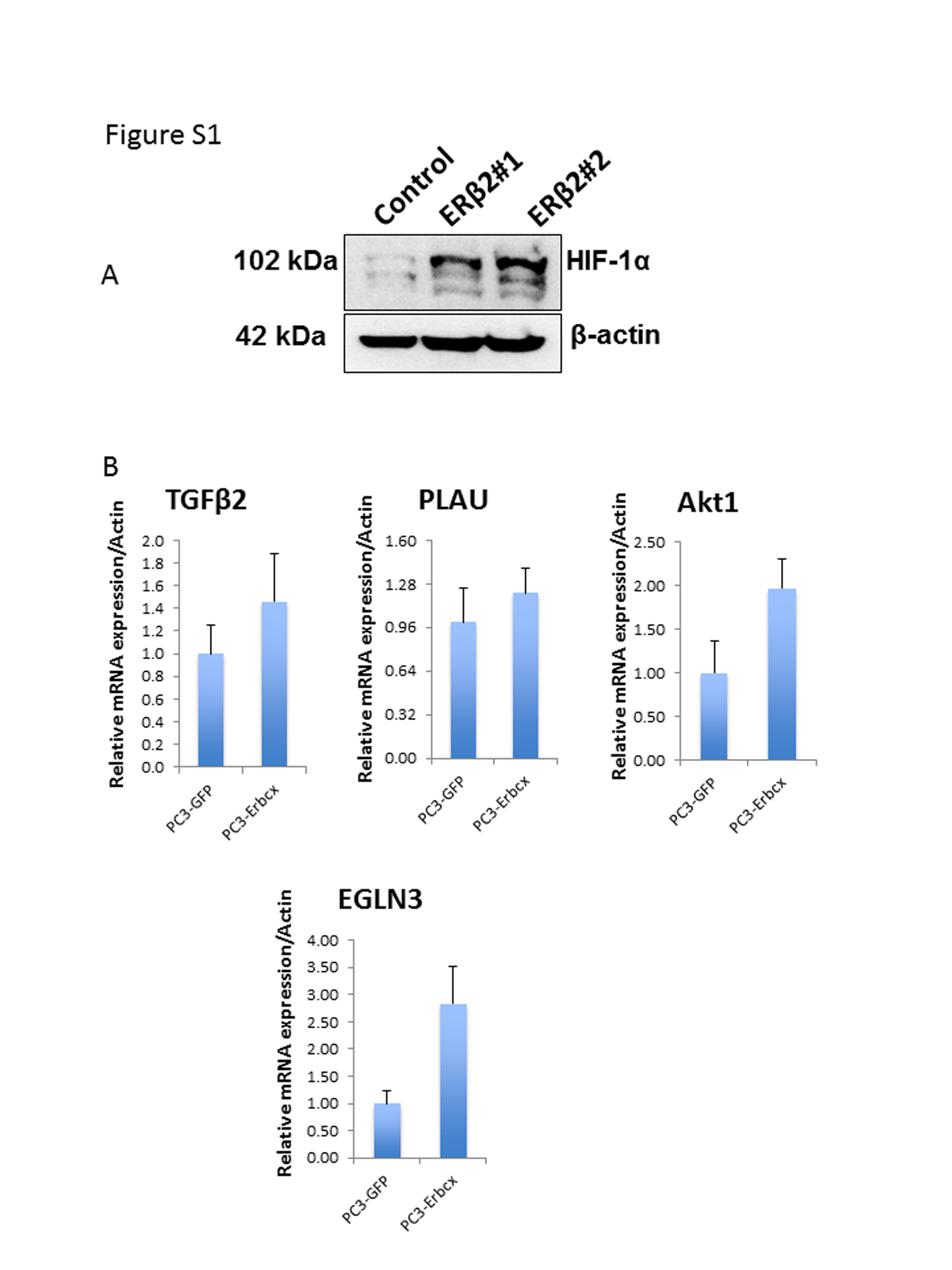

Supplement: S1 Fig — A. Western blot for HIF-1α on extracts from control 22Rv1 cells and two clones expressing ERβ2. (B) Validation of genes from the microarray. (TIF) [file pone.0128239.s001.tif]
